# Bibliographical Notices

**Published:** 1851-06

**Authors:** 


### BIBLIOGRAPHICAL NOTICES.

*New Remedies: with Formulæ for their administration.* By Robley Dunglison, M. D., Professor of the Institutes of Medicine, &c., in the Jefferson Medical College of Philadelphia. *Sixth Edition, with extensive additions.* 8vo. pp. 755. Philadelphia. Lea & Blanchard.

In noticing the sixth edition of so popular a work as the "New Remedies," it is sufficient for us to enumerate the additions which have been made to it since the publication of the fifth edition. "The last few years," as Dr. Dunglison well observes, "have been rich in valuable gifts to Therapeutics;" and in the following list of therapeutical agents, now introduced into the work, will be recognised some of the most valuable and approved remedial resources in actual use:—Adamsonia digitata, Benzoate of Ammonia, Valerianate of Bismuth, Sulphate of Cadmium, Chloroform, Collodion, Cantharidal Collodion, Cotyledon Umbilicus, Sulphuric Ether, Strong Chloric Ether, Compound

Ether, Hura Braziliensis, Iberis Amara, Iodic Acid, Iodide of Chloride of Mercury, Powdered Iron, Citrate of Magnetic Oxide of Iron, Citrate of Iron and Magnesia, Sulphate of Iron and Alumina, Tannate of Iron, Valerianate of Iron, Nitrate of Lead, Lemon Juice, Citrate of Magnesia, Salts of Manganese, Oleum Cadinum, Arsenite of Quinia, Hydriodate of Iron and Quinia, Sanicula Marilandica, and Sumbul.

An Index of Diseases, to which the remedies are applicable, has also been added to this edition, and will be found useful.

---

*Report of the Committee on the Statistics of Calculous Disease in Ohio, made to the Ohio State Medical Society.* By E. H. Davis, M. D.

This pamphlet presents the results obtained by the author, in an attempt to determine the influence of geological formations in the State of Ohio on the development of calculous diseases. The State of Ohio being nearly equally divided between the blue and cliff limestone formations on the west, and the sandstone or coal-bearing series on the east, affords an excellent field for the accumulation of facts bearing upon this interesting point. Dr. Davis has been able to collect reports from portions of the State, containing rather more than half its population, the average period of observation being fifteen years. They show a striking preponderance of calculous diseases in the limestone region. The proportion of cases here is found to be "1 case per annum to about 60,000 inhabitants," while "in the sandstone and coal series, we have 1 case per annum to 238,000 population, showing the disease to be nearly four times more frequent in the limestone formation than in the coal series."

Dr. Davis is led to infer that the *blue* limestone district is most favorable to the appearance of calculous concretions—although he deems his reports scarcely numerous enough to warrant positive conclusions in this particular. The blue limestone formation of Ohio is the same as that of Lexington, in Kentucky, the residence of Dr. Dudley, who, we believe, has performed the operation of lithotomy some two hundred times—a fact that strongly supports the inference of Dr. Davis as to the comparative frequency of calculous affections in the district in question.

Dr. Davis hopes to be able to extend his valuable statistics on this interesting hygienic question, and invites the fuller cöoperation of the profession both in Ohio and throughout the entire valley of the Mississippi. Communications on the subject are to be hereafter directed to him at New York.

---

*Intermarriage, or the mode in which, and the causes why, Beauty, Health and Intellect, result from certain unions, and deformity, disease and insanity, from others. Demonstrated by delineations of the structure and forms and description of the functions and capacities which each parent, in every pair, bestows on children—in conformity with certain natural laws, and by an account of corresponding effects in the breeding of animals. With eight illustrative drawings.* By ALEXANDER WALKER. Philadelphia: Lindsay & Blakiston. 1851.

The lengthy title given above almost serves the purpose of a table of contents to a work, whose main object is the announcement and illustration of a natural law. According to this law, one parent gives to the progeny the forehead and organs of sense, together with the nutritive organs contained within the trunk; while the other gives the backhead and cerebellum, (or organ of the will, as the author calls it,) together with the locomotive organs, comprising the exterior of the trunk and the whole of the limbs.

The author is evidently a careful observer and a profound thinker, and has presented us with a vast amount of information derived both from man and the inferior animals. He has aimed to be useful by pointing out how bodily deformities and mental infirmities may be forestalled; and how marriage among blood relations (which have always appeared to us a violation of the laws of nature) tends to the degeneracy of the offspring. He also shows how, by carefully assorted marriages, the means of improving general organization and beauty of countenance, as well as mental and physical vigor, are, in a great degree, under the control of man; this is familiar to every cattle breeder, who knows that breeding in-and-in, as it is called, is always attended by degeneracy in the offspring. Although not strictly a medical work, we cannot refrain from commending to the perusal of the profession the little volume of Mr. Walker, as containing much that is valuable in a hygienic point of view.

---

*Minor Surgery; or Hints on the Every-day Duties of the Surgeon.* By Henry H. Smith, M. D.; Assistant Lecturer on Clinical Surgery in the University of Pennsylvania, &c. &c. Third Edition, with numerous additions; illustrated by 247 engravings. Philadelphia: Barrington & Haswell, 1850.

Readiness and promptitude of action are among the most valuable attributes of the skilful surgeon, one of whose distinguishing characteristics is the dexterity with which he accommodates himself to circumstances, and bends them to his own immediate wants. To all who would obtain these desirable qualifications we commend the little work whose title stands at the head of this notice. It offers itself to the student and practitioner in its third edition, with much additional matter in the shape of descriptions of the duties of assistants at operations; the mode of conducting etherization; the mechanical treatment of club feet; the cure of aneurism by compression; the catoptric diagnosis of cataract; and similar subjects likely to prove of daily utility to the practitioner. It is well printed and handsomely gotten up.

---

*Quarterly Summary of the Transactions of the College of Physicians.* Philadelphia; Lippincott, Grambo & Co. Nov. 1850, to Jan., 1851, inclusive, and Jan. to April, 1851, inclusive.

We are pleased to welcome the Transactions in their new dress, as well as to learn that arrangements have been made to give them a more extended circulation. A new series is commenced with the Quarterly No. for January, which will be issued regularly, and may be obtained from the publishers at the low rate of one dollar per annum. The Nos. before us are handsomely printed, and illustrated with lithographic and wood engravings, and contain the medical reports, essays and debates, that occurred during the sessions of the college in the quarter prior to their publication.

*On the Theory and Practice of Midwifery.* By FLEETWOOD CHURCHILL, M. D., M. R. I. A., &c. &c. With notes and additition by D. FRANCIS CONDIE, M. D. Secretary of the College of Physicians, &c. &c. With one hundred and thirty-nine illustrations. A new American from the last improved Dublin Edition. Philadelphia; Blanchard & Lea. 1851.

We take great pleasure in announcing to our readers a new and improved edition of this standard work on obstetrics, made more valuable by the notes and additions of Dr. Condie, whose past experience in this department entitles him, justly, to be heard as an authority. Dr. Churchill's works are so well and so favorably known in this country, as to make more than a bare announcement of a new edition unnecessary. It is gotten up in the usual handsome style of the publishers, and will prove a welcome and faithful friend to the profession.

---

*Medical Commencement of the University of Pennsylvania, with a Valedictory Address*, by W. E. HORNER, M. D., Professor of Anatomy; April 5th, 1851.

*Charge to the Graduates of Jefferson Medical College of Philadelphia*, by T. D. MÜTTER, M. D., Professor of Surgery; March 8th, 1851.

*Charge to the Graduates of the Medical Department of Pennsylvania College*, by W. DARRACH, M. D., Professor of the Theory and Practice of Physic; March 6th, 1851.

The season of "commencements" is over, and the flocks of graduates are distributing themselves over the wide spread face of the country, freighted with the good advice and kind wishes of their quondam teachers! What conscientious and upright men they would be, did they but act up to the counsels so lavishly bestowed upon them! But, alas, the "*auri sacra fames*," and the jealousies of professional success, too often make *self* more prominent in the mind, than the great mission on which they have embarked.

Some few extracts from these various addresses, above named, are all that our limited space will allow, but these will serve to show the sageness of the counsel, and the warmth of feeling with which they abound.

"Indolence in the early years of professional life is by all means to be avoided; a pre-occupation of the public attention by seniors and by men of acknowledged skill, will necessarily leave the young practitioner in obscurity and without much reward. Many men are irrevocably discouraged by their deep sense of these disadvantages, and as they cannot get an immediate reward, have not the force of character to labor for the emoluments which come in, only after the expiration of ten or of fifteen years. The difference between individuals is strongly marked in this respect, because there are some who, notwithstanding this tedious postponement, yet consent to labor on, and to do so even diligently, under a strong hope that their reward must at length come. In surveying the state of our profession here, elsewhere in the cities of the United States, and in the largest of cities in Europe, as London and Paris, it will be found that the most distinguished and able members of the time are those choice spirits who in early life devoted days and nights to study; and to threading obscure alleys, the abodes of sickness and of wretched poverty; but having now passed through that probation, they are enjoying all the advantages and dignity of their high calling."

Speaking of the subject of medical education, and the conflict of opinion on this subject, Dr. Horner remarks:

"I am not prophet enough to foresee the final result of this struggle of antagonisms, but I trust it may be propitious to the cause of science. I at least have the consolation of representing here to-day an Institution which, the first in the country in its period of organization, has also led the way in the efforts to advance medical education—and while other institutions decline going counter to a popular current, is herself endeavoring to stem it, and, if possible, to give it a better direction. Whether we are to succeed or to fail in this effort, time has yet to determine. We would cordially invoke a greater number of sister schools to lay aside their fears and to co-operate with us. There will, I have no doubt, be a temporary sacrifice to them, as with us, but most probably an increased benefit will subsequently result. A sense of discrimination by the public may finally prevail, so as to understand that while there are many institutions having the right of issuing diplomas, so there must be other guarantees of proper educetion besides the mere exhibition of a parchment."

Again, in relation to short courses, and the influence that the profession at large is able to exert upon the community, he justly observes:

"We have heard of a medical education for California. Suited, however, as California may be for rapid growth of every kind, we doubt much whether what may suit California, will also be acceptable everywhere else. Ample as that market may be for doctors of all kinds, it is probable that it will soon be overstocked, and as all who wish an education for it, cannot be accommodated there, the overstock may be in some difficulty to find a congenial soil elsewhere. But, watchful observers exist even there, in the persons of experienced and educated physicians, who, to a large extent, influence the public mind, and determine more or less upon questions of personal merit."

The following brief notice of himself, as a matter of medical history, possesses interest to all to whom the distinguished author is known:

"With this address I close a circle of forty courses of lectures in the University of Pennsylvania. The forty years which they represent, have been in every way the eventful ones of my life: they include education, business, wife, children and friends. My connection with this distinguished school was first as a student; your partialities, too little merited, elevated me at an early period of life, to the honorable distinction of a place in its medical faculty. I became finally the successor in full of a Shippen, a Wistar, and a Physick, names too illustrious to need, at the present moment, further notice. I now find myself, though not very old, yet at a period of life when most men may look for retirement at a day not distant; either from the necessities of health, or in view of making place for the deserving who are close upon their steps. As this is moreover the last time in which I am likely, in the regular order of rotation, to perform an office similar to the present, I will take this only opportunity of speaking strongly, but with all respect to you.

"Let me then say, I trust that at no period, either while I am with you, or subsequently, the high principles which have hitherto governed this school may be abated; or that an honor suited only to minds of the higher cast of intelligence, shall be permitted to become a simple commodity, whose price of time and of labor is to be regulated by the freedom with which the supply may come from other quarters. As we have heretofore survived the influence of the latter policy from various directions, ever since my first connection with you, so may you survive it long after I am gone."

The charge of Dr. Mütter is an eloquent exposition of the aids and appliances by means of which success in professional life is most generally obtained: "I mean *honorable* success, for the mere acquisition of notoriety, or the accumulation of fortune, may most readily be reached by the possession of qualities directly the reverse of those with which every conscientious and virtuous physician should be imbued." Among these are enumerated: *a thorough medical education; habits of reasoning and thinking; a reverence for the art; honesty of purpose; discretion, self-respect, self-reliance, industry and perseverance, charity, ambition and patriotism,* all of which are enforced in the glowing language which no one knows better how to use. One sentiment we wish were more generally practiced: "A good rule is to refuse a certificate to every patent medicine or instrument, for I hold that every honorable physician is bound to make public any discovery calculated to benefit his fellow man."

We have occupied so much space with the extracts given above, that but little is left, except to speak in terms of high commendation of the charge of Dr. Darrach, which urges the importance of other qualifications than mere medical attainments; these are medical discernment, compassion to the sick, medical devotion, and medical liberality. These are the jewels with which the armor of the physician is adorned, in the figurative language of the author. We present our thanks to each and all for the pleasure afforded in the perusal of their several addresses.

